# Effect of combined tobacco use and type 2 diabetes mellitus on prevalent fibrosis in patients with MASLD

**DOI:** 10.1097/HC9.0000000000000300

**Published:** 2023-10-27

**Authors:** Oluwafemi Balogun, Jeffrey Y. Wang, Emad S. Shaikh, Karine Liu, Stefania Stoyanova, Zoe N. Memel, Hayley Schultz, Lisa Mun, Jack Bertman, Cheryl A. Rogen, Maryam K. Ibrahim, Madeline Berschback, Eugenia Uche-Anya, Robert Wilechansky, Tracey G. Simon, Kathleen E. Corey

**Affiliations:** 1 Department of Medicine, Liver Center, Division of Gastroenterology, Massachusetts General Hospital, Boston Massachusetts, USA; 2 George Washington University School of Medicine, Washington D.C., 2001; 3 Harvard Medical School, Boston Massachusetts, USA; 4 Department of Medicine, Massachusetts General Hospital, Boston Massachusetts, USA; 5 University of California San Francisco Medical Center, San Francisco, California, USA; 6 Central Michigan University College of Medicine, Mt Pleasant, Michigan; 7 Clinical and Translational Epidemiology Unit (CTEU), Massachusetts General Hospital, Boston Massachusetts, USA

## Abstract

**Background:**

Several studies have investigated the independent effect of cigarette smoking or type 2 diabetes mellitus (T2DM) on MASLD. However, the interaction effect between tobacco consumption and T2DM on MASLD severity remains underexplored. In this study, we assessed the combined effect of tobacco use and T2DM on hepatic fibrosis in MASLD.

**Methods:**

We conducted a single-center retrospective cross-sectional analysis of eligible participants from the Mass General Brigham Fibroscan© database. The participants were divided into 3 groups: those with T2DM and a history of tobacco use (primary exposure group), those with T2DM but no history of tobacco use (secondary exposure group), and those without T2DM and no history of tobacco use (reference group). An additional model was developed, which included a fourth group, participants with a history of tobacco use but no T2DM. The likelihood of fibrosis was determined using a defined fibrosis-4 index cutoff value of 1.3. In addition, we computed the estimated marginal means for liver stiffness measurement and compared the values among the exposure groups. Bivariable and multivariable logistic regression models were used to explore the associations between the exposure groups and the risk for hepatic fibrosis.

**Results:**

Overall, 598 individuals were enrolled in the study. The bivariable logistic regression model revealed a significant independent association between T2DM, combined smoking and T2DM, and the outcome of interest, fibrosis. Age, sex, metabolic syndrome, aspirin use, statin use, hemoglobin A1C (A1C), and total bilirubin level were also significantly associated with fibrosis. In the adjusted fibrosis-4 multivariable model (comparing exposure groups to controls), cigarette smoking and T2DM interaction had higher odds of prevalent fibrosis (aOR, 3.04; 95% CI, 1.62–5.76), compared to those with T2DM alone (aOR 2.28; 95% CI, 1.37–3.85). The continuous liver stiffness measurement comparison across the exposure group showed an estimated marginal means of 6.26 (95% CL: 5.58–6.94), 7.54 (95% CL: 6.78–8.30), and 7.88 (6.78–8.99) for the reference group, T2DM only group, and tobacco-T2DM group, respectively. The diabetes-only group and the combined tobacco-T2DM group had statistically significant associations with liver stiffness measurement (*p* values: 0.013 and 0.014, respectively).

**Conclusion:**

Although diabetes is independently associated with hepatic fibrosis in patients with MASLD, the combination of tobacco consumption and diabetes is associated with a higher prevalence of fibrosis. Therefore, lifestyle change through tobacco use cessation in patients with diabetes could be beneficial in reducing the incidence of liver fibrosis among individuals with MASLD.

## BACKGROUND

MASLD is one of the most common chronic liver diseases in the United States and globally, with a rising prevalence that is estimated to be 30% globally.^1^ MASH, a phenotype on the MASLD spectrum, can promote progression to fibrosis, cirrhosis, and potentially hepatocellular cancer.^2,3^ MASH cirrhosis is a leading cause of chronic liver disease and liver transplantation among adult patients in the United States.^4^ Studies show that while MASLD and MASH contribute to disease progression, fibrosis is the most significant predictor of liver-related outcomes and all-cause mortality.^5,6^ However, the drivers of fibrosis have not been extensively elucidated.

Insulin resistance and type 2 diabetes mellitus (T2DM) have been linked to the incidence and progression of MASLD.^7,8^ They have also been identified as critical components in the multicausal disease model for incident MASH and progression to fibrosis.^7,9^ Persistent hyperglycemia, secondary to uncontrolled or poorly controlled T2DM, promotes chronic glucotoxicity.^10^ By extension, hyperglycemia fosters the progression of hepatic steatosis, necroinflammation, and hepatocellular dysfunction.^7^

Similarly, tobacco consumption has been independently implicated in the incidence and progression of MASLD in some studies.^11–13^ The enhancing effect of tobacco use on MASH-associated and MASLD-associated fibrosis has been linked to insulin resistance and the release of pro-inflammatory cytokines. Conversely, other studies showed no clear relationship between tobacco use and MASLD.^14–16^ In addition to these conflicting findings, there is a paucity of evidential information on the association between smoking and MASLD fibrosis.

Several studies have investigated measures of effect and association between cigarette smoking, T2DM, and MASLD.^11,12,17^ However, the impact of the interaction between tobacco consumption and T2DM on MASLD severity remains unexplored. Identifying modifiable risk factors on the background of a baseline genetic susceptibility is crucial for MASLD management. In this study, we evaluated the association between tobacco use and T2DM interaction on MASLD fibrosis.

## METHODS

### Study design and population

We conducted a single-center cross-sectional analysis of patients diagnosed with MASLD. Adult patients who were 18 years and above, who underwent a FibroScan© (vibration-controlled transient elastography), and laboratory information available at the time of FibroScan assessment were included in the study. The FibroScan evaluation was performed by trained medical personnel. Study participants were required to fast for 2 hours before the procedure. During the procedure, the patients were laid supine, and the trained medical personnel applied an ultrasound-like probe on the skin over the region of the liver. FibroScan measurements were subsequently obtained. All scans with an interquartile range >30% were discarded, and scans with less than 10 accurate measurements were also rejected. MASLD was defined by the presence of diffuse fatty infiltration (increased echogenicity) on abdominal imaging, the appearance of macrovesicular fat accumulation in ≥5% of hepatocytes on histology, or FibroScan controlled attenuation parameter (CAP) score ≥248 dB/m.^18^ Patients with a history of excessive alcohol consumption (more than 7 drinks per week for women and more than 14 drinks per week for men), HIV, and other causes of chronic liver disease were excluded. The study’s main objective was to determine whether T2DM and tobacco use act synergistically to increase the odds of fibrosis in MASLD. Our primary outcome was fibrosis assessed via fibrosis-4 (FIB-4) scores^19^ and liver stiffness measurement (LSM) using Fibroscan.

The 2 outcomes were further recategorized into binary variables. LSM was dichotomized into stages 0–2 and stages 3–4 (“advanced fibrosis”) using a cutoff value of ≥x11.4 kPa. FIB-4 scores were computed using validated parameters (alanine transaminase, aspartate transaminase, age at FibroScan, and platelet count) and stratified into “no fibrosis” and “fibrosis,” with a defined cutoff score of 1.30.^20^ In addition, a FIB-4 cutoff value of 2.67 was chosen to rule in significant fibrosis. We tested associations with possible predictors^20^ and evaluated associations with secondary outcomes in the form of exploratory analyses. Hepatic steatosis was classified into mild-advanced and moderate-advanced steatosis (using a CAP cutoff value of ≥268).^18,21^ The crude means of numerical CAP, LSM, and FIB-4 scores were compared between the 3 primary exposure groups [reference (No T2DM, no tobacco), T2DM, and T2DM-tobacco]. For comparative purposes, we also included a “Tobacco-only” group in a separate table and compared the parameter means with the original exposure groups. Additional adjustments for confounding variables were included in a multivariate logistic regression model to assess the adjusted means between the 3 groups.

### Data extraction and patient characteristics

Data extracted from the electronic medical record included relevant demographic, clinical, and laboratory information. The collated information was stored in the Mass General FibroScan© Database. Parameters included in the database are date of birth, race, body mass index (BMI), date of FibroScan, biopsy date, history of diabetes, history of hypertension, medication use (statin, aspirin, glucagon-like peptide-1 analogs, metformin, histamine H2 receptor blockers, proton pump inhibitors, angiotensin receptor blockers, angiotensin-converting enzyme inhibitors), mortality, hepatocellular cancer occurrence, tobacco use, alcohol consumption, liver disease etiology, liver enzymes, lipid profile, and blood glucose assessment tests. History of diabetes mellitus was extracted from the electronic medical records and defined as the use of antidiabetic medications for the management of hyperglycemia, fasting plasma glucose ≥x126 mg/dL, hemoglobin A1C ≥6.5, random plasma glucose or post–2-hour oral glucose tolerance test ≥x200 mg/dL.

Due to a low number of active tobacco users, tobacco use was categorized into “never smoker” and “ever smoker” (comprised of both former and current smokers). BMI, a continuous variable, was stratified into participants with <25 kg/m^2^ (normal and reference category), patients between 25 and 29.9 kg/m^2^ (overweight), and individuals with a BMI ≥30 kg/m^2^ (obese). The decision to group underweight and normal-weight individuals into the same group (less than 25 kg/m^2^) was based on the evidence of only 1 participant with a BMI of less than 18.5 kg/m^2^. Aspirin, statin, metformin, Glucagon-Like Peptide 1 (GLP-1) analogs, PPIs, H2 blockers, and angiotensin-converting enzyme use were categorized into “current use” and “no current use.” Alcohol use history was recorded as a continuous variable indicating the number of alcohol-associated drinks consumed weekly.

### Statistical analysis

Descriptive and inferential statistics were computed to reveal the baseline characteristics and explore the associations between exposure groups and multiple outcomes, respectively. Continuous data are presented as mean (unless otherwise stated) and the corresponding SD (±SD). Categorical variables are expressed in counts and percentages. Bivariable logistic regression models were developed to evaluate the independent effects of the variables of interest on fibrosis (through LSM-stratified and FIB-4-stratified parameters). Measures of effect were reported using OR, their corresponding 95% CI, and accompanying *p* values. We also computed false discovery rate as q-values (set at a significant alpha value of 0.05) into the models to account for multiple comparisons. This adjustment was incorporated in subsequent exploratory analyses. Secondary analyses were performed to compare the means of CAP, LSM, and FIB-4 scores (as continuous numerical variables) and to determine the differences between the exposure groups. Furthermore, discrete multiple linear regression models were developed to explore the role primary exposure played in the observed secondary outcomes while adjusting for potential confounding variables.

### Sensitivity and exploratory analyses

Sensitivity analyses were performed to investigate the primary outcomes (degree of steatosis and fibrosis) with defined cutoff values of 1.45 to rule out fibrosis and 3.25 to rule in advanced fibrosis^22^ ^(p4)^. In addition, we performed exploratory analyses to assess other predictors of fibrosis and to explore independent associations between expanded exposure groups (comprising reference (no T2DM, no tobacco), T2DM, tobacco, and T2DM-tobacco), clinical variables of interest, and the likelihood of fibrosis (assessed using LSM and FIB-4 dichotomized values) among the study cohort. A cutoff value of ≥7.9 kPa and ≥10 kPa was employed to rule in fibrosis and advanced fibrosis, respectively, using the LSM, and a cutoff score of ≤1.45 was chosen to rule out fibrosis using the FIB-4 score.^23,24^ A nominal, 2-sided, *p* ≤0.05 was considered statistically significant.

## RESULTS

### Population characteristics

**FIGURE 1:**
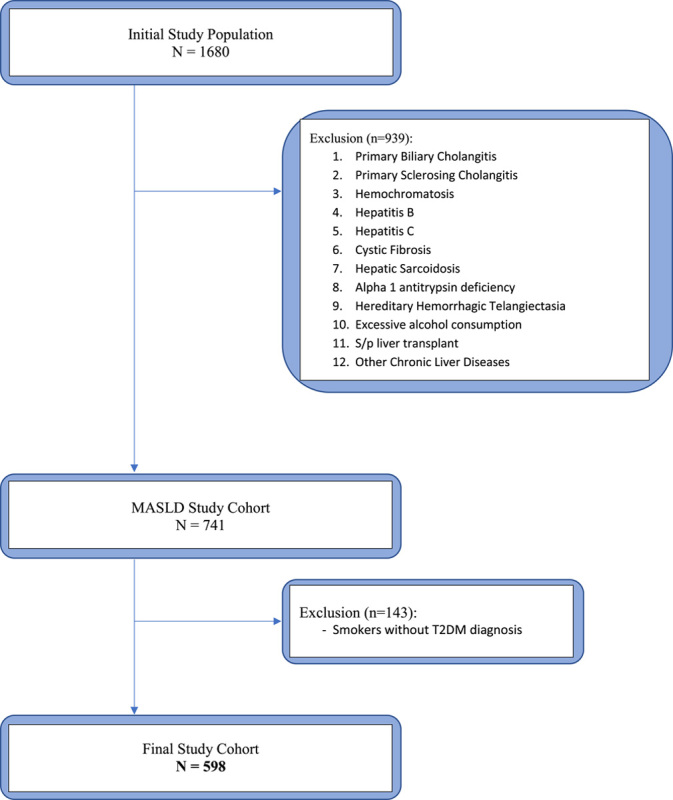
Flowchart demonstrating the eligibility for study participation. Abbreviations: MASLD, metabolic dysfunction-associated liver disease; T2DM, type 2 diabetes mellitus.

A total of 560 eligible participants were enrolled in the study (Figure 1). Of the eligible cohort, 97 (16.2%) had T2DM-tobacco (primary exposure group), 206 (34.4%) had T2DM (secondary exposure group), and 257 (43%) had no history of T2DM or tobacco use (reference group). Most participants were female (334; 56%) and white (410; 69%). The average age of the study cohort was 56, and the mean BMI was 33 kg/m^2^(Table 1). A diagnosis of hypertension was present in 328 (55%) participants and dyslipidemia was evident in 346 (58%) patients. Further stratification by exposure group revealed that the average age for combined T2DM-Tobacco history, T2DM, and reference group [no T2DM, no tobacco] were 65 years, 57 years, and 53 years, respectively. Hypertension diagnosis was similar across the primary (89%) and secondary exposure groups (81%) but lower in the reference group (30%). Other baseline characteristics are summarized in Table 1 and Supplemental Table 1, http://links.lww.com/HC9/A602.

**TABLE 1:** Demographics and baseline characteristics of patients enrolled in the study

Characteristics	No T2DM, no tobacco (N = 400)	T2DM (N = 206)	T2DM + tobacco (N = 97)
Age (Mean/SD, y)			
Mean (SD)	54 (± 14)	57 (± 13)	65 (± 10)
Age category (y), n (%)			
< 35	36 (9)	10 (5)	1 (1)
35–49	103 (26)	44 (21)	5 (5)
50–64	159 (40)	90 (44)	39 (40)
> 65	102 (26)	62 (30)	52 (54)
Sex (female), n (%)	223 (56)	131 (64)	57 (59)
Race, n (%)			
Asian	29 (7)	14 (7)	3 (3)
Black	6 (2)	10 (5)	1 (1)
Hispanic	6 (2)	17 (8)	2 (2)
Others	46 (12)	30 (15)	7 (7)
White	313 (78)	134 (65)	84 (87)
Body mass index (Mean/SD, kg/m2)	32 (± 6.4)	33 (± 6.2)	34 (± 6.1)
Hypertension, n (%)	133 (33)	166 (81)	86 (89)
Dyslipidemia, n (%)	149 (37)	164 (80)	90 (93)
Liver stiffness measurement (Mean/SD, kPa)	6.5 (± 4.5)	7.5 (± 6.6)	7.9 (± 5.5)
Controlled attenuation parameter (Mean/SD, CAP)	310 (± 64)	310 (± 68)	310 (± 61)
Antihypertensives use^a^, n (%)	109 (27)	88 (43)	45 (46)
ALT (Mean/SD)	44 (± 31)	40 (± 27)	41 (± 26)
AST (Mean/SD)	34 (± 20)	33 (± 18)	35 (± 20)
A1C (Mean/SD)	5.8 (± 1.1)	6.5 (± 1.4)	6.6 (± 1.3)
Total bilirubin (Mean/SD)	0.61 (± 0.38)	0.57 (± 0.39)	0.53 (± 0.30)
Aspirin use, n (%)	105 (26)	71 (34)	48 (49)
Statin use, n (%)	150 (38)	123 (60)	70 (72)
GLP analog use, n (%)	13 (3)	21 (10)	16 (16)
Metformin, n (%)	63 (16)	91 (44)	51 (53)
PPI use, n (%)	126 (32)	70 (34)	45 (46)
H2 blockers, n (%)	36 (9)	14 (7)	7 (7)

a Antihypertensives—angiotensin-converting enzyme inhibitor use or angiotensin II receptor blocker use.

Abbreviations: A1C, glycated hemoglobin; ALT, alanine transaminase; AST, aspartate transaminase; GLP, glucagon-like peptide; H2, histamine 2; PPI, proton pump inhibitor.

### Bivariable associations

The primary exposure group (T2DM-tobacco vs. reference [no T2DM, no tobacco]) was significantly associated with the likelihood of fibrosis (assessed using FIB-4 scores, *p* < 0.001) in unadjusted models. In addition, the covariates of age, BMI, sex, hypertension, dyslipidemia, and certain medications (aspirin, statin, angiotensin-converting enzyme, and angiotensin receptor blockers) were independently and significantly associated with the presence of fibrosis (*p* < 0.050, Table 2). In unadjusted models, individuals with combined T2DM-tobacco exposure were 3.5 times more likely to have prevalent fibrosis than the reference group (OR: 3.50 95% CI: 2.15, 5.79). Participants with T2DM were 1.9 times more likely to have prevalent fibrosis when compared with the reference group (OR: 1.88, 95% CI: 1.29, 2.75; Table 2). Additionally, participants in xthe Tobacco group were 1.6 times more likely to have prevalent fibrosis when compared to the reference group (OR 1.61, 95% CI: 1.03, 2.51).

**TABLE 2:** Bivariable model revealing the independent associations between baseline characteristics and outcome of interest (likelihood of fibrosis using FIB-4)

Characteristic	N	Event N	OR^***1***^	95% CI^***1***^	*P*
Exposure group	551	246	—	—	**<0.001**
No T2DM, no tobacco (ref)	—	—	—	—	—
T2DM	—	—	1.88	1.29, 2.75	—
T2DM + tobacco	—	—	3.50	2.15, 5.79	—
Age	551	246	1.12	1.10, 1.14	**<0.001**
BMI	549	245	0.97	0.94, 0.99	**0.013**
Sex (female, ref)	551	246	1.62	1.15, 2.29	**0.006**
Hypertension	551	246	3.62	2.52, 5.25	**<0.001**
Dyslipidemia	551	246	2.73	1.90, 3.94	**<0.001**
GLP analog use	550	246	1.56	0.82, 3.01	0.18
Combined aspirin–statin use^a^	551	246	6.57	4.25, 10.4	**<0.001**
Antihypertensive use^b^	549	244	1.83	1.29, 2.62	**<0.001**

a Use of aspirin, statin, or both.

b Use of ACEi, ARB, or both.

Abbreviations: ACEIs, angiotensin-converting enzyme inhibitors; ARBs, angiotensin receptor blockers; BMI, body mass index.

Note: Bold values denote P<0.05

### (Multivariable associations)

After adjusting for the effect of potentially confounding variables, T2DM-tobacco was associated with significantly higher odds of fibrosis when compared with the reference group (OR: 1.88, 95% CI: 1.04, 3.43, *p*-value = 0.0037). Similarly, the T2DM group was also associated with increased odds of fibrosis when compared with the reference group (OR: 1.13, 95% CI: 0.70, 1.80, *p* = 0.6). However, the magnitude of the effect was less than the primary exposure group, and the statistical association was not significant (Table 3). BMI, coexisting hypertension, and sex were all significantly associated with fibrosis (*p* < 0.050). The use of metformin and GLP analog were both associated with decreased odds of fibrosis, OR: 0.72 and OR: 0.63, respectively. However, the association was not statistically significant (*p* = 0.200 and *p* = 0.300). In another multivariable logistic regression model that included the Tobacco group, participants in the Tobacco group had 1.8 times the odds of prevalent fibrosis (compared to the reference group) assessed using LSM-derived fibrosis (Table 4). In contrast, the T2DM group and combined T2DM-tobacco group both had an OR of 1.58 (0.90–2.76) and 2.13 (1.25–3.65).

**TABLE 3:** Measures of effect and associations between main exposure groups and the likelihood of prevalent fibrosis by FIB-4 while adjusting for possible confounding variables (multivariable model)

Characteristic	OR	95% CI	*P*
Exposure group			
No T2DM, no tobacco (ref)	—	—	—
T2DM	1.13	0.70, 1.80	0.6
T2DM + tobacco	1.88	1.04, 3.43	**0.037**

Abbreviation: T2DM, type 2 diabetes mellitus.

Note: Bold values denote P <0.05

**TABLE 4:** Multivariable model exploring the associations between exposure groups (with tobacco-only group inclusive) and LSM-derived hepatic fibrosis

Characteristic	OR	95% CI	*P*
Exposure			
Reference	—	—	
T2DM	1.58	0.90, 2.76	0.11
T2DM + tobacco use	2.13	1.25, 3.65	0.006
Tobacco	1.80	0.94, 3.44	0.073

Abbreviation: T2DM, type 2 diabetes mellitus.

### Bivariable and multivariable associations using LSM as a surrogate marker of fibrosis

The continuous LSM comparison across the exposure group showed an estimated marginal means of 6.26 kPa (95% CL: 5.58–6.94), 7.54 kPa (95% CL: 6.78–8.30), and 7.88 kPa (6.78–8.99) for the reference group, T2DM group, and T2DM-tobacco group, respectively. The T2DM group and T2DM-tobacco group had statistically significant associations with LSM (*p* values: 0.013 and 0.014, respectively).

In the bivariable model, there was a significant relationship between the exposure groups and the outcome. T2DM were 1.8 times more likely to have prevalent advanced fibrosis, while participants with T2DM who used tobacco had 2.4 times the odds of advanced fibrosis compared to control (Supplemental table 2, http://links.lww.com/HC9/A602). In the same model, age, metabolic syndrome, statin and aspirin use, aspartate transaminase levels, and platelet count were also significantly associated with prevalent advanced fibrosis. In the multivariable model, the T2DM group had 1.8 times the odds of advanced fibrosis, and the T2DM + tobacco exposure group was 2.3 times more likely to have advanced fibrosis (Table 5). However, these associations were not statistically significant (*p* = 0.082 and *p* = 0.052, respectively).

**TABLE 5:** Multivariable model showing the associations between exposure groups and fibrosis (assessed with LSM)

Characteristic	OR	95% CI	*P* a
Exposure group			
No T2DM, no tobacco (ref)	—	—	—
T2DM only	1.83	0.93, 3.64	0.082
T2DM + tobacco use	2.27	0.99, 5.19	0.052

a Adjusted for age, sex, dyslipidemia, and platelet count.

Abbreviation: T2DM, type 2 diabetes mellitus.

### Exploratory analyses

The independent association between the expanded exposure groups (Supplemental Tables 2, 3, & 4, http://links.lww.com/HC9/A602) and fibrosis (estimated using LSM > 7.9, FIB-4 > 1.45, and LSM > 10, respectively) was statistically significant (*p* < 0.001). Further evaluation of the individual components of the composite exposure group (LSM > 7.9) revealed that the tobacco-use-only group were 1.6 times more likely to have fibrosis, the T2DM group had 2.4 times the probability of prevalent fibrosis, and the combined T2DM + Tobacco participants were 2.2 times more likely to have fibrosis when compared to controls. The associations between the newly expanded exposure groups and fibrosis (assessed using FIB-4 scores > 1.45) showed a similar trend with the *p* values (Supplemental Table 4, http://links.lww.com/HC9/A602). However, the T2DM-Tobacco group had higher odds of prevalent fibrosis than other nonreference groups.

## DISCUSSION

This study offers new insights into the roles of diabetes and tobacco use in predicting hepatic fibrosis among patients with MASLD and how these 2 modifiable factors compound the risk of fibrosis progression. While T2DM and smoking are established risk factors for liver fibrosis and MASLD, respectively, the combined effects of tobacco use and T2DM on liver fibrosis had not previously been well defined. The present study demonstrates that smoking and underlying T2DM have a synergistic effect on the severity of fibrosis compared to nonsmokers with T2DM. Similar to prior findings, our study revealed that individuals diagnosed with T2DM had a greater prevalence of MASLD-associated hepatic fibrosis.^25,26^ Barb et al similarly reported a 2-fold increase in prevalent MASLD-related fibrosis with T2DM, though the study was limited to overweight and obese individuals. In addition, the study observed that individuals in the T2DM-tobacco group were 3 times more likely to have the outcome of interest. This finding implies that tobacco use with a background of diabetes results in an additional 1-fold increase in the probability of fibrosis. Thus, while it has been shown that tobacco use and T2DM are independent risk factors for MASLD severity, the additive effect of both risk factors was demonstrated in our study. We explored this concept to delineate the cumulative effects of tobacco use on a background of T2DM in comparison to healthy controls and nonsmokers with T2DM.

Although the precise pathological mechanism for the observed additive effect of combined T2DM and tobacco use remains unclear, we propose 2 possible explanations. First, evidence supports the independent and direct effect of tobacco use on insulin resistance.^27^ This association may be mediated by adiponectin, a secretory adipokine produced by the adipocytes and significant in regulating lipid metabolism and fatty acid oxidation.^9^ Adiponectin has also been shown to negatively correlate with insulin resistance, with lower levels observed among tobacco users.^28^ In addition to the adiponectin effect, smoking has also been indirectly linked to insulin resistance through visceral adiposity. Canoy et al revealed in their study a significant association between cigarette smoking and visceral abdominal adiposity.^29^ A higher measure of central obesity was significantly observed among tobacco users. This association suggests a stronger link between insulin resistance and hepatic fibrosis among patients with MASLD. The second possible explanation for our findings could be that tobacco use creates direct hepatotoxic effects on the liver. For example, there is a known increase in specific pro-inflammatory cytokines (IL-1, IL-6, IL-8, and TNF- α) associated with tobacco use.^30,31^ These cytokines promote oxidative stress, enhance activation of stellate cells, and subsequently result in liver fibrosis.^31^ In a higher-risk substrate already predisposed to hepatic steatosis and insulin resistance (such as someone with T2DM), the inflammatory damage tobacco causes may serve as a catalyst for accelerated fibrosis.

In addition, the study’s data concerning the prevalence of dyslipidemia and hypertension among patients with MASLD with T2DM and tobacco use serve as a distinct reminder of the substantial cardiovascular disease risks faced by this population. The coexistence of T2DM and smoking with these additional risk factors in patients with MASLD significantly amplifies their vulnerability to cardiovascular disease-related morbidity and mortality. To address the morbidity and mortality associated with cardiovascular disease in patients with MASLD, a multidisciplinary approach is essential, encompassing not only liver health but also comprehensive cardiovascular risk assessment and management. This holistic approach can ultimately lead to improved outcomes and a better quality of life for individuals living with MASLD. Health care providers can also offer comprehensive support to patients with MASLD with the dual burdens of T2DM and tobacco use while simultaneously reducing the morbidity and mortality associated with cardiovascular disease in this high-risk population.

There are several limitations to this study. First, our research was observational, and causal inference cannot be determined. Also, fibrosis was noninvasively assessed using the FIB-4 index and FibroScan. Liver biopsy is currently the gold standard for the histological confirmation of liver fibrosis31. Furthermore, there was limited data on the number of pack-years of tobacco use. Hence, the dose-response relationship between smoking and fibrosis could not be assessed. Also, the study population was predominantly white. Further observational studies on a more diverse population would be beneficial for generalization purposes. Lastly, data on waist circumference and waist-to-height ratio were not available. The absence of these markers of central adiposity precluded the estimation of visceral fat accumulation.

In conclusion, the findings of this study provide concrete risk estimates for clinicians working to counsel patients with MASLD on smoking cessation and optimizing diabetes control. Based on our findings, we believe that smoking cessation, in addition to standard of care, may be beneficial in reducing the severity of MASLD among patients with T2DM.

## Supplementary Material

SUPPLEMENTARY MATERIAL
