# Supplementary material for: Effect of combined tobacco use and type 2 diabetes mellitus on prevalent fibrosis in patients with MASLD
Source: Hepatol Commun. 2023 Oct 27;7(11):e0300. doi: 10.1097/HC9.0000000000000300 (PMC10615418; doi:10.1097/HC9.0000000000000300)
Supplement: SUPPLEMENTARY MATERIAL [file hc9-7-e0300-s001.docx]

**Table 1. Demographics and baseline characteristic of patients enrolled in the study**

| **Characteristics** | **No T2DM, No Tobacco (**N=400**)** | **T2DM (**N=206**)** | **T2DM+Tobacco (**N=97**)** |
| --- | --- | --- | --- |
| **Age (**Mean/SD, years**)** |  |  |  |
| Mean (SD) | 54 (± 14) | 57 (± 13) | 65 (± 10) |
| **Age Category (years)** |  |  |  |
| < 35 | 36 (9 %) | 10 (5 %) | 1 (1 %) |
| 35 – 49 | 103 (26 %) | 44 (21 %) | 5 (5 %) |
| 50 – 64 | 159 (40 %) | 90 (44 %) | 39 (40 %) |
| >65 | 102 (26 %) | 62 (30 %) | 52 (54 %) |
| **Gender (**Female**)** | 223 (56 %) | 131 (64 %) | 57 (59 %) |
| **Race** |  |  |  |
| Asian | 29 (7 %) | 14 (7 %) | 3 (3 %) |
| Black | 6 (2 %) | 10 (5 %) | 1 (1 %) |
| Hispanic | 6 (2 %) | 17 (8 %) | 2 (2 %) |
| Others | 46 (12 %) | 30 (15 %) | 7 (7 %) |
| White | 313 (78 %) | 134 (65 %) | 84 (87 %) |
| **Body Mass Index (**Mean/SD, kg/m2**)** | 32 (± 6.4) | 33 (± 6.2) | 34 (± 6.1) |
| **Hypertension** | 133 (33 %) | 166 (81 %) | 86 (89 %) |
| **Dyslipidemia** | 149 (37 %) | 164 (80 %) | 90 (93 %) |
| **Liver Stiffness Measurement (**Mean/SD**,** kPA**)** | 6.5 (± 4.5) | 7.5 (± 6.6) | 7.9 (± 5.5) |
| **Controlled Attenuation Parameter (**Mean/SD**,** CAP**)** | 310 (± 64) | 310 (± 68) | 310 (± 61) |
| **Antihypertensives Use^a^** | 109 (27 %) | 88 (43 %) | 45 (46 %) |
| **ALT (**Mean/SD) | 44 (± 31) | 40 (± 27) | 41 (± 26) |
| **AST (**Mean/SD) | 34 (± 20) | 33 (± 18) | 35 (± 20) |
| **A1C (**Mean/SD) | 5.8 (± 1.1) | 6.5 (± 1.4) | 6.6 (± 1.3) |
| **Total Bilirubin (**Mean/SD) | 0.61 (± 0.38) | 0.57 (± 0.39) | 0.53 (± 0.30) |
| **Aspirin Use** | 105 (26 %) | 71 (34 %) | 48 (49 %) |
| **Statin Use** | 150 (38 %) | 123 (60 %) | 70 (72 %) |
| **GLP Analogue Use** | 13 (3 %) | 21 (10 %) | 16 (16 %) |
| **Metformin** | 63 (16 %) | 91 (44 %) | 51 (53 %) |
| **PPI Use** | 126 (32 %) | 70 (34 %) | 45 (46 %) |
| **H2 Blockers** | 36 (9 %) | 14 (7 %) | 7 (7 %) |

^a^Antihypertensives – angiotensin converting enzyme inhibitor use or angiotensin II receptor blocker use.

ALT – Alanine transaminase, AST – Aspartate transaminase, A1C – Glycated hemoglobin, GLP – Glucagon-like peptide, PPI – Proton pump inhibitor, H2 – Histamine 2

**Figure 1. Flow chart demonstrating eligibility for study participation**

Exclusion (n=143):

- Smokers without T2DM diagnosis

Final Study Cohort

**N = 598**

MASLD Study Cohort

N = 741

Exclusion (n=939):

1. Primary Biliary Cholangitis
2. Primary Sclerosing Cholangitis
3. Hemochromatosis
4. Hepatitis B
5. Hepatitis C
6. Cystic Fibrosis
7. Hepatic Sarcoidosis
8. Alpha 1 antitrypsin deficiency
9. Hereditary Hemorrhagic Telangiectasia
10. Excessive alcohol consumption
11. S/p liver transplant
12. Other Chronic Liver Diseases

Initial Study Population

N = 1680

**Table 2. Bivariable model revealing the independent associations between baseline characteristics and outcome of interest (likelihood of fibrosis using FIB4)**

| **Characteristic** | **N** | **Event N** | **OR***^1^* | **95% CI***^1^* | **p-value** |
| --- | --- | --- | --- | --- | --- |
| **Exposure Group** | 551 | 246 |  |  | **<0.001** |
| No T2DM, No Tobacco (ref) |  |  | — | — |  |
| T2DM |  |  | 1.88 | 1.29, 2.75 |  |
| T2DM+Tobacco |  |  | 3.50 | 2.15, 5.79 |  |
| **Age** | 551 | 246 | 1.12 | 1.10, 1.14 | **<0.001** |
| **BMI** | 549 | 245 | 0.97 | 0.94, 0.99 | **0.013** |
| **Gender (**female, ref**)** | 551 | 246 | 1.62 | 1.15, 2.29 | **0.006** |
| **Hypertension** | 551 | 246 | 3.62 | 2.52, 5.25 | **<0.001** |
| **Dyslipidemia** | 551 | 246 | 2.73 | 1.90, 3.94 | **<0.001** |
| **GLP Analogue Use** | 550 | 246 | 1.56 | 0.82, 3.01 | 0.18 |
| **Combined Aspirin-Statin Use^a^** | 551 | 246 | 6.57 | 4.25, 10.4 | **<0.001** |
| **Antihypertensives Use^b^** | 549 | 244 | 1.83 | 1.29, 2.62 | **<0.001** |
| ^s^Use of aspirin, statin, or both  ^b^Use of ACEi, ARB, or both | | | | | |

**Table 3. Measures of effect and associations between main exposure groups and likelihood of prevalent fibrosis by FIB4, while adjusting for possible confounding variables (Multivariable Model)**

| **Characteristic** | **OR***^1^* | **95% CI***^1^* | **p-value** |
| --- | --- | --- | --- |
| Exposure Group |  |  |  |
| No T2DM, No Tobacco (ref) | — | — |  |
| T2DM | 1.13 | 0.70, 1.80 | 0.6 |
| T2DM+Tobacco | 1.88 | 1.04, 3.43 | **0.037** |
|  | | | |
| ^1^ OR = Odds Ratio, CI = Confidence Interval | | | |

**Table 4. Multivariable model showing the associations between exposure groups and fibrosis (assessed with LSM)**

| **Characteristic** | **OR***^1^* | **95% CI***^1^* | **p-value**^2^ |  | |
| --- | --- | --- | --- | --- | --- |
| **Exposure Group** |  |  |  |  | |
| No T2DM, No Tobacco (ref) | — | — |  |  | |
| T2DM Only | 1.83 | 0.93, 3.64 | 0.082 |  | |
| T2DM+Tobacco Use | 2.27 | 0.99, 5.19 | 0.052 |  | |
|  |  |  |  |  | |
| *^1^*OR = Odds Ratio, CI = Confidence Interval  *^2^*Adjusted for age, gender, dyslipidemia, platelet count | | | | |  |

**Table 5. Multivariable model exploring the associations between exposure groups (with tobacco-only group inclusive) and LSM-derived hepatic fibrosis.**

| **Characteristic** | **OR***^1^* | **95% CI***^1^* | **p-value** |
| --- | --- | --- | --- |
| **Exposure** |  |  |  |
| Reference | — | — |  |
| T2DM | 1.58 | 0.90, 2.76 | 0.11 |
| T2DM + Tobacco Use | 2.13 | 1.25, 3.65 | 0.006 |
| Tobacco | 1.80 | 0.94, 3.44 | 0.073 |
|  | | | |
| ^1^ OR = Odds Ratio, CI = Confidence Interval | | | |

**Supplementary Information (Tables and Figures)**

**Supplementary Table 1. Baseline characteristics of study participants with additional information on ‘Tobacco Only’ group.**

|  | **Control Group (N=257)** | **Tobacco Only (N=143)** | **Diabetes Only (N=206)** | **T2DM+Tobacco (N=97)** |
| --- | --- | --- | --- | --- |
| **Age (years)** |  |  |  |  |
| Mean (SD) | 53 (± 14) | 58 (± 13) | 57 (± 13) | 65 (± 10) |
| **Gender** |  |  |  |  |
| Female | 146 (57 %) | 77 (54 %) | 131 (64 %) | 57 (59 %) |
| **Body Mass Index (kg/m2)** |  |  |  |  |
| Mean (SD) | 32 (± 6.5) | 33 (± 6.2) | 33 (± 6.2) | 34 (± 6.1) |
| **Hypertension** |  |  |  |  |
| HTN | 30% | 40% | 81% | 89% |
| **Liver Stiffness Measurement (kPA)** |  |  |  |  |
| Mean (SD) | 6.3 (± 4.6) | 6.9 (± 4.4) | 7.5 (± 6.6) | 7.9 (± 5.5) |
| **Controlled Attenuation Parameter (CAP)** |  |  |  |  |
| Mean (SD) | 300 (± 67) | 310 (± 59) | 310 (± 68) | 310 (± 61) |
| **ALT** |  |  |  |  |
| Mean (SD) | 43 (± 31) | 45 (± 32) | 40 (± 27) | 41 (± 26) |
| **AST** |  |  |  |  |
| Mean (SD) | 33 (± 17) | 36 (± 25) | 33 (± 18) | 35 (± 20) |
| **LDL** |  |  |  |  |
| Mean (SD) | 110 (± 38) | 110 (± 40) | 99 (± 40) | 95 (± 40) |
| **HDL** |  |  |  |  |
| Mean (SD) | 52 (± 16) | 51 (± 17) | 49 (± 18) | 48 (± 14) |
| **Triglycerides** |  |  |  |  |
| Mean (SD) | 150 (± 87) | 180 (± 120) | 190 (± 250) | 180 (± 140) |
| **Total Cholesterol** |  |  |  |  |
| Mean (SD) | 190 (± 41) | 190 (± 45) | 180 (± 55) | 180 (± 45) |
|  |  |  |  |  |

**Supplementary Table 2. Bivariable model showing the associations between key clinical variables and fibrosis (assessed with LSM)**

| **Characteristic** | **N** | **Event N** | **OR***^1^* | **95% CI***^1^* | **p-value** | **q-value***^2^* |
| --- | --- | --- | --- | --- | --- | --- |
| **Exposure Group** | 559 | 66 |  |  | **0.031** | **0.062** |
| No T2DM, No Tobacco (ref) |  |  | — | — |  |  |
| T2DM Only |  |  | 1.78 | 0.98, 3.27 |  |  |
| T2DM+Tobacco Use |  |  | 2.39 | 1.19, 4.75 |  |  |
| **Age** | 559 | 66 | 1.02 | 1.00, 1.04 | **0.022** | **0.050** |
| **Gender (**female, ref**)** | 559 | 66 | 0.95 | 0.56, 1.60 | 0.86 | 0.87 |
| **Metabolic Syndrome** | 555 | 66 | 3.06 | 1.73, 5.69 | **<0.001** | **<0.001** |
| **Aspirin Use** | 559 | 66 | 2.04 | 1.20, 3.44 |  | **0.027** |
| **Statin Use** | 559 | 66 | 2.37 | 1.39, 4.17 | **0.001** | **0.006** |
| **Total Bilirubin** | 555 | 65 | 1.06 | 0.51, 1.99 | 0.87 | 0.87 |
| **ACEi Use** | 559 | 66 | 2.10 | 1.20, 3.62 |  | **0.028** |
| **ARB Use** | 557 | 65 | 1.15 | 0.53, 2.28 |  | 0.80 |
| **H2 Blockers** | 559 | 66 | 0.52 | 0.12, 1.50 | 0.25 | 0.37 |
| **PPI Use** | 546 | 63 | 1.22 | 0.70, 2.09 |  | 0.60 |
| *^1^*OR = Odds Ratio, CI = Confidence Interval  *^2^*False discovery rate correction for multiple testing  ACEi – Angiotensin-converting enzyme inhibitor, ARB- Angiotensin II receptor blocker, H2 – Histamine 2, PPI – Proton pump inhibitor | | | | | | |

**Supplementary Table 3. Bivariable association between exposure groups and likelihood of fibrosis using Liver Stiffness measurement (with cutoff value of 7.9kPA)**

| **Characteristic** | **N** | **Event N** | **OR***^1^* | **95% CI***^1^* | **p-value** | **q-value***^2^* |
| --- | --- | --- | --- | --- | --- | --- |
| **Exposure Group** | 702 | 157 |  |  | **<0.001** | **0.002** |
| Reference |  |  | — | — |  |  |
| Tobacco Use |  |  | 1.60 | 0.94, 2.70 |  |  |
| T2DM |  |  | 2.44 | 1.55, 3.88 |  |  |
| T2DM+Tobacco Use |  |  | 2.22 | 1.26, 3.89 |  |  |
| **Age** | 739 | 165 | 1.01 | 1.00, 1.02 | 0.19 | 0.24 |
| **BMI** | 700 | 158 | 1.12 | 1.09, 1.15 | **<0.001** | **<0.001** |
| **Gender (**female, ref**)** | 731 | 164 | 0.84 | 0.59, 1.20 | 0.34 | 0.34 |
| **Hypertension** | 739 | 165 | 1.74 | 1.22, 2.49 | **0.002** | **0.004** |
| *^1^*OR = Odds Ratio, CI = Confidence Interval  *^2^*False discovery rate correction for multiple testing | | | | | | |

**Supplementary Table 4**. Sensitivity analysis of the bivariable associations between exposure groups and likelihood of fibrosis using FIB4 scores (with a cutoff value of 1.45)

| **Characteristic** | **N** | **Event N** | **OR***^1^* | **95% CI***^1^* | **p-value** | **q-value***^2^* |
| --- | --- | --- | --- | --- | --- | --- |
| **Exposure** | 692 | 245 |  |  | **<0.001** | **<0.001** |
| Reference |  |  | — | — |  |  |
| Tobacco Use |  |  | 1.61 | 1.03, 2.51 |  |  |
| T2DM |  |  | 1.87 | 1.26, 2.79 |  |  |
| T2DM+Tobacco Use |  |  | 2.91 | 1.78, 4.76 |  |  |
| **Age** | 728 | 260 | 1.12 | 1.10, 1.14 | **<0.001** | **<0.001** |
| **BMI** | 693 | 245 | 0.95 | 0.93, 0.98 | **<0.001** | **<0.001** |
| **Gender (female, ref)** | 720 | 254 | 1.18 | 0.87, 1.61 | 0.29 | 0.29 |
| **Hypertension** | 728 | 260 | 2.76 | 2.01, 3.82 | **<0.001** | **<0.001** |
| *^1^*OR = Odds Ratio, CI = Confidence Interval  *^2^*False discovery rate correction for multiple testing | | | | | | |

**Supplementary Table 5. Multivariable model exploring the associations between exposure groups (with tobacco-only group inclusive) and LSM-derived hepatic fibrosis (LSM>10).**

| **Characteristic** | **OR***^1^* | **95% CI***^1^* | **p-value** |
| --- | --- | --- | --- |
| **Exposure** |  |  |  |
| No T2DM, No Tobacco (ref) | — | — |  |
| T2DM | 1.50 | 0.85, 2.64 | 0.2 |
| T2DM + Tobacco Use | 2.27 | 1.31, 3.98 | 0.004 |
| Tobacco | 1.83 | 0.92, 3.63 | 0.086 |
|  | | | |
| ^1^ OR = Odds Ratio, CI = Confidence Interval | | | |
